# Ethnic Differences in Context: Does Emotional Conflict Mediate the Effects of Both Team- and Individual-Level Ethnic Diversity on Emotional Strain?

**DOI:** 10.1007/s41542-021-00105-5

**Published:** 2022-03

**Authors:** Franziska J. Kößler, Kaori Fujishiro, Susanne Veit, Annekatrin Hoppe

**Affiliations:** 1 Department of Psychology, Humboldt-Universität zu Berlin, Rudower Chaussee 18, 12489 Berlin, Germany; 2 Graduate Program ‘Good Work’, WZB Berlin Social Science Center, Berlin, Germany; 3 Division of Field Studies and Engineering, National Institute for Occupational Safety and Health, Centers for Disease Control and Prevention, Cincinnati, Ohio, USA; 4 German Center for Integration and Migration Research (DeZIM-Institut), Berlin, Germany; 5 Research Unit ‘Migration, Integration, Transnationalization’, WZB Berlin Social Science Center, Berlin, Germany

**Keywords:** Ethnic heterogeneity, Ethnic dissimilarity, Well-being, Status, Relational demography, Social processes

## Abstract

Work teams are becoming increasingly heterogeneous with respect to their team members’ ethnic backgrounds. Two lines of research examine ethnic diversity in work teams: The compositional approach views team-level ethnic heterogeneity as a team characteristic, and relational demography views individual-level ethnic dissimilarity as an individual member’s relation to their team. This study compares and contrasts team-level ethnic heterogeneity and individual-level ethnic dissimilarity regarding their effects on impaired well-being (i.e., emotional strain) via team- and individual-level emotional conflict. Fifty teams of retail chain salespeople (*n* = 602) participated in our survey at two points of measurement. Based on the ethnic background of team members, we calculated team-level ethnic heterogeneity that applied to all members, and individual-level ethnic dissimilarity within the team that varied according to each member’s ethnic background. Multilevel path modeling showed that high levels of team-level ethnic heterogeneity were related to high levels of emotional strain via team-level emotional conflict. However, the opposite was found for individual-level ethnic dissimilarity. We discussed this difference by contextualizing individual-level ethnic dissimilarity in the team-level heterogeneity and social status of ethnic groups in society at large. Our findings suggest that the social status of the ethnic group to which team members belong may impact how ethnic diversity relates to team processes and well-being.

Occupational Health Psychology has become increasingly important for individuals, organizations, and society in the last decades as we observe a worldwide rise in work-related mental illnesses (Harvey et al., 2017). Because workplaces reflect changes in society at large, resulting shifts in work environments may be contributing to this rise in poor mental health. One major societal change in recent decades is global migration. As the volume of migration increases, societies have become more ethnically diverse (United Nations, Department of Economic, & Social Affairs, Populations Division, 2018). Workplaces in receiving countries have also become ethnically diverse (International Labour Organization, 2017), which highlights the importance of investigating the effects of working with others with different ethnic backgrounds, especially on employee well-being.

Two lines of research have addressed ethnic diversity in organizations. The compositional approach has considered team-level ethnic heterogeneity as a team characteristic (i.e., a team’s ethnic composition; Harrison & Klein, 2007) and mostly focuses on team-level outcomes such as team performance (Joshi et al., 2011). Individual-level effects of ethnic diversity have been addressed in the literature of relational demography (Meyer, 2017). The focus is on how team members experience individual-level ethnic dissimilarity within their team (i.e., how similar or different a team member is from the rest of the team; Riordan, 2000) and how this, in turn, affects health and well-being. With these different levels of foci, the two lines of research have remained largely separate, with Brodbeck et al. (2011), Chatman and Flynn (2001), and Leonard and Levine (2006) being exceptions to this. These three studies provide first insights into how the team- and individual-level manifestations of ethnic diversity interact. However, they all focused on performance outcomes. Thus, it is not well understood how the combined effects of team-level ethnic heterogeneity and individual-level ethnic dissimilarity impact employee well-being.

In this study, we explore the effects of the two levels of ethnic diversity on individual employees’ emotional strain—a state of impaired well-being (Mohr et al., 2006)—with emotional conflict as a mediator at both levels. This study contributes to the literature on ethnic diversity in the following ways. First, by investigating team-level ethnic heterogeneity and individual-level ethnic dissimilarity together in real work teams, we explore potentially different effects of ethnic diversity at the team and individual levels. Second, by investigating the effects of individual-level ethnic dissimilarity in the context of team-level ethnic heterogeneity, we shed light on differences in the experience of emotional conflict between ethnic majority and minority workers. We discuss these differences in relation to ingroup/outgroup processes and status differences (Chattopadhyay et al., 2004; Tajfel & Turner, 1986). Finally, we focus on impaired well-being (i.e., emotional strain) as an individual-level outcome, which has not yet received much attention as an outcome of team-level ethnic heterogeneity. This recognition has important implications for creating work teams that support well-being of their members with diverse ethnic backgrounds. Because team processes in these ethnically heterogeneous work teams could be shaped by management practices and supervisor training, this study contributes to promoting employee well-being in today’s diverse workplaces.

## Ethnic Diversity in Organizations—Two Levels of Manifestation

The separate lines of research—one focusing on team-level ethnic heterogeneity, the other focusing on individual-level ethnic dissimilarity—limit our understanding of ethnic diversity in organizations. The team’s ethnic heterogeneity and each member’s ethnic dissimilarity with others are related but different constructs. Yet, both shape individuals’ experience of ethnic diversity simultaneously; that is, the team’s heterogeneity gives a dynamic context in which individual dissimilarity impacts well-being (Hoppe et al., 2014). Consider two five-person teams: a highly heterogeneous team with five ethnicities represented by one member each, and a more homogeneous team with four members of one ethnicity and one member of another. The ethnic minority member in the latter team and all members of the former team have the exact same level of individual ethnic dissimilarity, but the team contexts are vastly different. This contextual information could be crucial in understanding why some studies on individual-level ethnic dissimilarity have produced null results (e.g., Jehn et al., 1997). Focusing on one without considering the other leaves unanswered questions (Riordan & Shore, 1997; Riordan & Wayne, 2008).

Another reason to consider team-level ethnic heterogeneity and individual-level ethnic dissimilarity together is the potential to uncover cross-level effects (i.e., team-level ethnic heterogeneity on individual team members’ well-being). This is a logical extension of the literature because the same theories underpin both the team-level and individual-level consequences of ethnic diversity. According to the similarity/attraction paradigm (Byrne, 1971) and the social identity approach (combining the social identity theory by Tajfel & Turner, 1986 and the self-categorization theory by Turner et al., 1987), working with people who are dissimilar to oneself is emotionally taxing. Both theories assume that people categorize themselves and others as ingroup or outgroup members (i.e., social/self-categorization) based on salient characteristics (e.g., demographics; Turner et al., 1987). The social identity approach suggests that people categorize themselves and others to gain a stable identity but also that they view members of their ingroup as superior to others (Tajfel & Turner, 1986), which might result in ingroup favoritism as well as derogation towards the outgroup (i.e., intergroup bias; Brewer, 1979). Relational demography has applied these theories to explain the negative impacts of individual-level ethnic dissimilarity on well-being. Because these theories can also explain the adverse effects of team-level ethnic heterogeneity on group dynamics, an association with impaired well-being can also be expected. However, to the best of our knowledge, studies on team-level ethnic heterogeneity have not yet explored its relation to impaired well-being or health (Meyer, 2017).

Taken together, we argue that effects of high individual-level ethnic dissimilarity on impaired well-being must be understood in the context of team-level ethnic heterogeneity. To date, three studies examined team-level ethnic heterogeneity together with individual-level ethnic dissimilarity (Brodbeck et al., 2011; Chatman & Flynn, 2001; Leonard & Levine, 2006). However, these studies tested effects of ethnic diversity on job satisfaction and performance (e.g., learning, turnover, and team effectiveness) that are of interest mainly for organizations but not directly relevant to employee health and well-being. Nevertheless, we can draw on these studies as they showed that effects of ethnic diversity exist simultaneously at both levels and that they can be in opposite directions (Brodbeck et al., 2011; Leonard & Levine, 2006). For example, team-level ethnic heterogeneity was positively related to learning performance, and individual-level ethnic dissimilarity was negatively related to learning performance for ethnic minority students (Brodbeck et al., 2011). These findings underline that ethnic diversity is a multilevel phenomenon with complex cross-level effects. Another reason to draw on these studies is that they showed that social processes mediate the relationships between ethnic diversity and performance at both levels. For example, Chatman and Flynn (2001) showed that cooperation at both levels mediated the effect of ethnic diversity on performance outcomes.

## Emotional Conflict as Mediating Mechanism

Because the same theories underpin both lines of research, studies on team composition and relational demography have assumed that social processes such as emotional conflict mediate between ethnic diversity and outcomes. Emotional conflict in teams is defined as the perception of interpersonal discrepancies, mutual dislike, tensions, and negative feelings among team members (De Dreu & Weingart, 2003; Jehn & Mannix, 2001). Individual-level emotional conflict can be informed by a team member’s own experiences (i.e., being directly involved in emotional conflicts) or by second-hand experiences (i.e., witnessing or hearing about disagreements among other members). Team-level emotional conflict, a shared perception among team members (Klein & Kozlowski, 2000), is informed by individual team members’ perceptions but does not imply that *all* members have direct experiences (Tjosvold, 2008). Emotional conflict can be measured using a referent-shift consensus model, that is, individual team members report the extent to which they perceive emotional conflict in reference to their team. This measurement approach distinguishes team-level and individual-level portions of the phenomenon and allows the investigation of emotional conflict as a potential mediator of ethnic diversity at multiple levels (Chan, 1998).

## Emotional Conflict and Impaired Well-Being

Individual-level emotional conflict is a social stressor that is likely to cause impaired well-being (Spector & Bruk-Lee, 2008). Although evidence is scarce, team-level emotional conflict can also be associated with team members’ impaired well-being. Social contagion, that is, the transmission of emotions or moods from one person to another through social interactions and processes, might explain this cross-level relationship (Hatfield et al., 1994). Empirical support for this mechanism comes from a study of 55 work teams showing that team-level emotional conflict was positively associated with staff burnout (Leon-Perez et al., 2016)—a construct related to emotional strain.

**Fig. 1:**
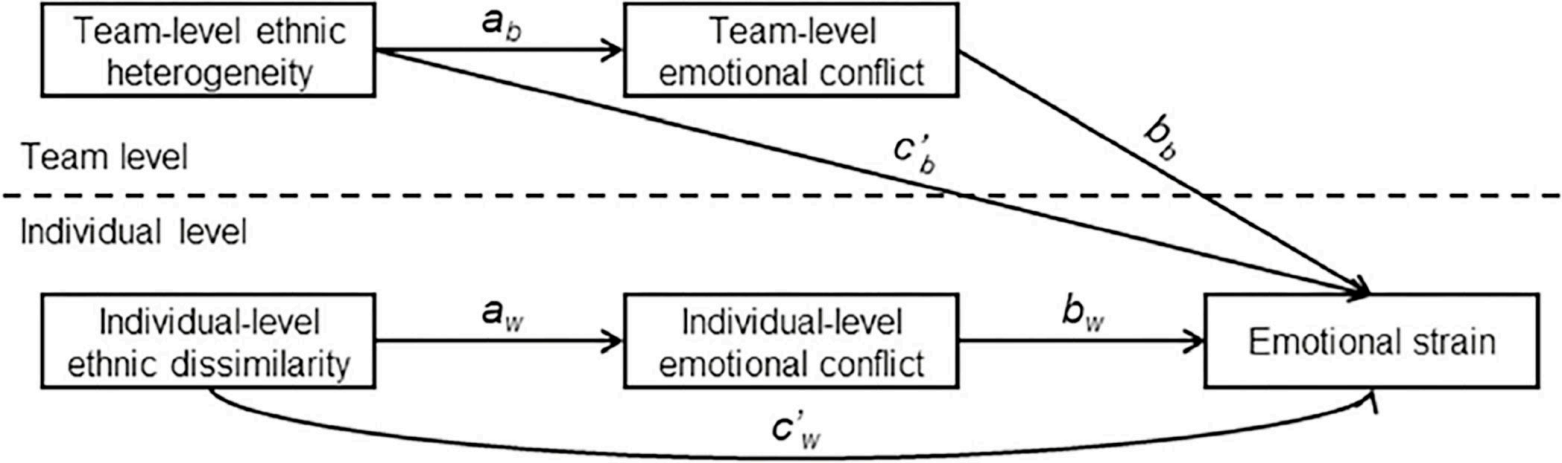
Conceptual Model. *Note*. The subscript _*b*_ indicates a path at the between-team level (i.e., team level); the subscript _*w*_ indicates a path at the within-team level (i.e., individual level)

We examined team- and individual-level emotional conflict as mediating mechanisms to better understand the relationship between ethnic diversity and impaired well-being. We chose emotional strain, which refers to the state of being easily irritated and quickly upset (Mohr et al., 2006), as the individual-level outcome. Although emotional strain is not sufficiently severe to be categorized as an illness, a longitudinal study has shown that emotional strain mediates between conflict and depressive symptoms (Dormann & Zapf, 2002). Taken together, we propose that emotional conflict mediates between ethnic diversity and emotional strain at two levels (see Fig. 1).

## Hypotheses

The theoretical rationale based on the social identity approach and the similarity/attraction paradigm has suggested that ethnically homogeneous work teams perceive less team-level emotional conflict than ethnically heterogeneous teams (e.g., Meyer, 2017; van Knippenberg et al., 2004). Studies conducted with different kinds of teams have provided empirical support for this relationship (e.g., Drach-Zahavy & Trogan, 2013; Pelled et al., 1999). Team-level emotional conflict as a social stressor has been suggested and shown to impair well-being at the team level (De Dreu & Weingart, 2003; de Wit et al., 2012). Combining the literature on team-level ethnic heterogeneity with the theoretical and empirical evidence that team-level emotional conflict impairs well-being, we hypothesize the following:

Hypothesis 1: The relationship between team-level ethnic heterogeneity and emotional strain will be mediated by team-level emotional conflict, such that higher levels of team-level heterogeneity are associated with higher levels of team-level emotional conflict, which, in turn, are associated with higher levels of emotional strain.

Based on a similar theoretical rationale, relational demography has suggested that ethnically dissimilar team members perceive higher levels of individual-level emotional conflict and have poorer social relationships with their team than ethnically similar members (Riordan, 2000). Guillaume et al. (2012) summarized empirical support for this suggestion in a meta-analysis. A broad range of theoretical and empirical literature has shown that individual-level emotional conflict at work is a social stressor that impairs well-being and health (e.g., De Dreu & Beersma, 2005; Dormann & Zapf, 2002; Spector & Jex, 1998). Based on relational demography and the association of individual-level emotional conflict with individual-level impaired well-being, we hypothesize the following:

Hypothesis 2: The relationship between individual-level ethnic dissimilarity and emotional strain will be mediated by individual-level emotional conflict, such that higher levels of individual-level ethnic dissimilarity are associated with higher levels of individual-level emotional conflict, which, in turn, are associated with higher levels of emotional strain.

## Methods

### Study Background

We gathered data from 50 work teams in a German retail chain in one major city. This specific study setting offered many advantages but also introduced some complications. In the following section, we provide some relevant details on the German context and work in a retail chain.

### Studying Ethnic Differences in Germany

Ethnic minority members^1^ who live in Germany are highly diverse. The first large-scale immigration in the 1960s primarily originated in Turkey and Mediterranean countries; later, immigrants from other European countries, the former USSR, and African and Middle Eastern countries came to Germany (Hansen, 2003). These waves of immigration have contributed to a highly diverse group of ethnic minorities now representing approximately 21% of the German workforce (Statistisches Bundesamt, 2018).

Despite the diversity in the ethnic minority population, collecting information about race in Germany is not feasible because of the racist crimes that occurred during the Third Reich and the use of the term “race” in that period (Berg et al., 2014). Instead of asking about racial identity from our study participants, we focused on ethnic background. Differences in ethnic background may entail differences in cultural knowledge, language skills, outer appearance, and names. Because manifestations of ethnic background vary widely, in this study we captured it in a broad way by asking about the ethnic origin of workers’ families, operationalized as birth country of workers and that of the parents (see also Measures section).

In Germany, children of immigrants face similar socioeconomic problems and prejudices to their parents (Hartmann, 2016). The history of migration in Germany sheds light on reasons for this situation: In the 1960s, people migrated to Germany through guest-worker programs, which tacitly assumed that these groups would ultimately return home. This assumption manifested in limited governmental efforts to properly integrate these workers and their families (Hansen, 2003). This governmental attitude is reflected in linguistic terms: Even today, immigrants and their German-born children are labeled “foreigners” (e.g., in labor-market statistics) or “people with a migration background/history” (Ohliger, 2008). Thus, in this study, we categorized both immigrants and their children as ethnic minority members (Statistisches Bundesamt, 2018).

### Retail Industry

Many ethnic minority members in Germany work in service jobs. In 2015, approximately 18% of the retail workforce consisted of ethnic minorities (Schäfer & Schmidt, 2016), a figure similar to the overall presence of ethnic minorities in the German workforce (Statistisches Bundesamt, 2018).

In retail stores, workers belong to clearly defined, physically separated, real work teams. In this study, the entire staff at each store location is defined as a team. To explore the working conditions in retail stores, the first author interned with two teams^2^ for two weeks and interviewed their members. The team members worked with all of their coworkers because the shift composition changed weekly. As expected, the contact between teams (i.e., across stores) was low because each team worked in a separate store. Therefore, the team was the most salient work unit.

All team members performed similar tasks, such as customer service, store maintenance, and working at the cash register. These tasks were highly standardized, which rendered task-related disagreements unlikely and helped us focus on emotional conflict. Team members worked on most of these tasks on their own, but when working next to each other, they talked to each other. Task distribution required coordination. In each team, a supervisor and an assistant coordinated shifts.

### Procedure

Data were collected from August to November 2017 through a self-administered questionnaire. The retail chain’s management encouraged the team members to participate in the study by crediting their participation time to their time accounts and allowing participation during regular work time. We provided the team members with small gifts. In exchange for supporting the study, management received a report on team members’ well-being in an aggregated form.

We informed team members about the purpose of the study and answered questions as we distributed the questionnaire in person during team meetings. Team members who were absent that day received an informational flyer and the questionnaire. Completed questionnaires were sent by post or put into a ballot box, which we retrieved after two weeks. Completing the questionnaire required 15 to 20 min.

We asked the team members to complete the survey at two time points with a time lag of one month. Both instances of the surveys included all study variables. We did not expect meaningful changes to occur during such a short period and thus did not consider this a longitudinal study. Rather, using self-reported data from different time points helped us minimize inflated associations among the study variables, which may be caused by transient moods or idiosyncratic events during the day (Podsakoff et al., 2003). The teams’ compositions remained relatively stable, and all team members worked with all others at least once over one month. Because we avoided the holiday season, the workload was also stable during this time.

An academic ethical committee and the retail chain’s works council approved the study. Participation in the survey was voluntary, and team members could contact us to withdraw from the study at any time using the contact details that we provided to them during survey distribution. To assure team members’ anonymity, we matched the first and second surveys of each team member using a personal code created by the team members themselves. Additionally, we assigned a code to each team, which was necessary for calculating ethnic diversity, but kept these code assignments separate from the surveys.

### Recruitment

We invited 50 teams to participate, all of which took part in the study. The team size, including the supervisor, ranged from 8 to 34 (*M* = 15.6, *SD* = 5.7). In the two data collection sessions held one month apart (T1 and T2), a total of 704 members had the opportunity to complete the questionnaire at least once, and 606 (86%) did so. Of the 98 nonparticipants, at least 43 were sick or on maternity leave and 12 were on vacation; for the rest the reasons were unknown. Of the 50 teams, 40 had a participation rate of 80% or higher, including 15 teams with a 100% participation rate. The team-level ethnic heterogeneity was not associated with the team’s participation rate (Kendall’s τ = .10, *p* = .34).

Of the 606 respondents, 428 (71%) provided data at both T1 and T2, 135 (22%) provided data only at Tl, and 43 (7%) provided data only at T2. Because we used emotional conflict data from Tl and the emotional strain data from T2, 43 members had missing data for the mediator measure, and 135 had missing data for the outcome measure. Full-information maximum likelihood estimation was used to account for the missing data. Four respondents were excluded from the analysis because they answered only the demographic questions and neither the mediator nor the outcome questions.

### Sample

Among all team members, 37% were 30 years old or younger, 37% were between 31 and 50 years old, and 16% were 51 years old or older. Most team members were women (91%). On average, team tenure was approximately five years (*SD* =4.9). Twenty-four different ethnic backgrounds were represented in our sample, and 17% of the members had an ethnic background other than German. This proportion of ethnic minorities is similar to that in the overall German retail workforce (Schäfer & Schmidt, 2016).

Half of the ethnic minority team members were born outside of Germany, and the other half were born in Germany to immigrant parents. The major ethnic backgrounds represented in our sample were Turkish (24%), Russian (23%), and Polish (13%). Regarding languages, more than half of the ethnic minority team members spoke a language other than German as their first language (62%), 30% spoke German as their first language, and 8% were raised in a bilingual household with German and another language.

Ethnic majority and minority team members were demographically similar, with two exceptions: Compared to ethnic majority team members, the ethnic minority members were less likely to have a vocational degree (86% vs. 62%), *X*^*2*^(1) = 28.92, *p* <.001, and to hold managerial positions (49% vs. 35%), *Z* =−3.40, *p* < .001. Because of the differences in responsibilities, ethnic majority team members worked more hours per week (*M* = 27.53, *SD* = 8.38) than ethnic minority team members (*M* = 24.86, *SD* = 9.09), *t* (576) = 2.76, *p* = .006.

### Measures

During the preparation phase of the study, we conducted cognitive interviews (Prüfer & Rexroth, 2010) to ensure that all team members understood the survey questions. As most customer contact and all written communication from management were in German, we assumed that the team members had sufficient German language skills. Thus, the survey was offered only in German, but we provided assistance during survey completion if necessary. We adapted the wording and the question order based on observations and interviews with team members of different ages, gender, and ethnic backgrounds. For means, standard deviations, Cronbach’s α, and test-retest reliability (*r*_*tt*_) see Table 1.

### Ethnic Background, Team-Level Ethnic Heterogeneity, and Individual-Level Ethnic Dissimilarity

#### Ethnic Background

To determine each team member’s ethnic background, we asked about the birthplace of their parents. If both parents were born in the same country (91%), this country was selected as the team member’s ethnic background. If one parent was born in Germany and the other parent was born abroad (4%), we assigned the ethnic background of the foreign-born parent. If both parents were from different foreign countries (1%), the mother’s birthplace was assigned (Constant et al„ 2012).^3^

#### Team-Level Ethnic Heterogeneity

To operationalize team-level ethnic heterogeneity, we followed Harrison and Klein (2007) and calculated Blau’s index (*BI*; Blau, 1977) for each team. In $BI=1-\sum p_{k}^{2},p$ refers to the proportion of team members with a particular ethnic background, and k refers to the number of ethnicities in the team. For example, calculating *BI* for a team of five Turkish members, ten Germans, and one Russian results in $BI=1-\left[{\left(5/16\right)}^{2}+{\left(10/16\right)}^{2}+{\left(1/16\right)}^{2}\right]=.51$. If all team members had the same ethnic background, BI was zero. The value of *BI* increases asymptotically—depending on the team size, the number of ethnic groups in the team, and the ethnic groups’ proportions in the team—to the theoretical maximum of one (Agresti & Agresti, 1978). In our sample, *BI* ranged from .00 to .73.

#### Individual-Level Ethnic Dissimilarity

To operationalize individual-level ethnic dissimilarity, we calculated the proportional dissimilarity (PD) for each team member as the proportion of team members whose ethnicity differed from the focal team member’s ethnicity (Williams and Meân, 2004), that is, $PD=n_{\text{dis}\;}/\left(n_{\text{team}\;}-1\right)$, where $n_{\text{dis}}$ is the number of ethnically dissimilar coworkers, and $n_{\text{team}}$ -1 is the total number of coworkers. In a team with five Turkish members, ten German members, and one Russian member, the Turkish members’ PD=(10+1)/(16−1)=.73, the German members’ PD=(5+1)/(16−1)=.40, and the Russian member’s PD=(5+10)/(16−1)=1.00. The resulting continuous variable can range from zero (i.e., all coworkers share the focal team member’s ethnic background) to one (i.e., no coworkers share the focal member’s ethnic background). In our study, PD ranged from zero (n=108) to one (n=80).

Calculating team-level ethnic heterogeneity and individual-level ethnic dissimilarity required information on all team members’ ethnic backgrounds regardless of their study participation (Allen et al., 2007; Riordan, 2000). If the information was missing for some team members either because they did not disclose this information in the survey or because they did not participate at all, we used administrative data provided by management. We were thus able to calculate team-level ethnic heterogeneity of all 50 teams and individual-level ethnic dissimilarity of all team members.

#### Emotional Conflict

We translated and back-translated an English emotional conflict scale (Jehn, 1994; Pelled et al., 1999) into German.^4^ The team members answered to four items (e.g., “Personal problems exist in our team.”) on a Likert scale ranging from 0 (*I disagree*) to 4 (*I fully agree*). Previous studies have shown that this scale can be aggregated to measure team-level emotional conflict, i.e., shared perceptions of emotional conflict in the team (Pelled et al., 1999), or to capture individual-level emotional conflict, i.e., individual perceptions (Jehn, 1994). Thus, we used this measure to operationalize emotional conflict at both levels. As shown in Table 1, team- and individual-level emotional conflict are stable over one month.

#### Emotional Strain

We measured emotional strain with the emotional subscale of the irritation scale by Mohr et al. (2006). Team members replied to three items such as “I react irritated although I do not intend to do so” using a Likert scale ranging from 0 (*I disagree*) to 4 (*I fully agree;* see also Hoppe, 2011). We excluded two items from the original five-item subscale of emotional irritation. In a cross-cultural validation by Mohr et al. (2006), these two items were shown to be culturally sensitive, and excluding them improved the factorial validity of the irritation scale and did not affect its good reliability. In our sample, emotional strain is stable (Table 1).

#### Socio- and Occupational Demographics

We asked the team members about their ages (in 10-year steps), gender (*male/female*), team tenure in years, their current position within the team (e.g., supervisor), their weekly working hours, and whether they had a vocational degree (*yes/no)*. In addition to their parents’ birthplaces, we asked all team members about their own birthplace, first language, citizenship, and the number of years they had lived in Germany.

### Statistical Analysis

To examine the indirect effects at two levels, we applied a multilevel path analysis in *Mplus* version 8.3. Data preparation was performed in *RStudio* version 1.1.456. As our data were hierarchically structured with individual team members nested in teams, we tested our hypotheses in a model with a random intercept. Additionally, the intraclass correlations (ICCs) of emotional conflict and emotional strain supported this decision because they lay within or close to the range that Bliese (2000) considered to be typical for team research (see Table 1). We selected a maximum likelihood estimator with robust Huber-White standard errors to address nonnormality.

We tested both hypotheses in one model and specified similar models with team-level ethnic heterogeneity and individual-level ethnic dissimilarity as predictors, team- and individual-level emotional conflict as mediators, and emotional strain as an individual-level outcome.^5^ Thus, we tested similar *a-, b-,* and *c’-*paths at both levels and calculated the indirect effects. Furthermore, we followed the recommendations by Preacher et al. (2010) on multilevel mediation analysis and decomposed the variance of emotional conflict and emotional strain, which were measured by individual-level self-reports, into latent between- and within-team variance. For testing indirect effects, we applied a Monte Carlo approach (Preacher & Selig, 2012) and derived asymmetric Monte Carlo-confidence intervals (MC-CIs) from an online calculator (Selig & Preacher, 2008).

According to Hypothesis 1, high team-level ethnic heterogeneity is related to high team-level emotional conflict (*a*_*b*_-path), which is related to high emotional strain (*b*_*b*_-path). The direct effect of team-level ethnic heterogeneity on emotional strain was included in the model (*c*’_*b*_-path, see Fig. 1). Together, these paths represent a 2-2-1 mediation (Preacher et al., 2010). We specified all of these effects at the team level using the latent between-team variance components of emotional conflict and emotional strain. We measured emotional strain for the individual. However, it is not possible to predict individual-level variance using a team-level predictor (Preacher et al., 2010; Zhang et al., 2009), so relationships between Level-2 predictors and Level-1 outcomes must be modeled as Level-2 relationships.

According to Hypothesis 2, high individual-level ethnic dissimilarity is related to high individual-level emotional conflict (*a*_*w*_-path), which is related to high emotional strain (*b*_*w*_-path). The direct effect of individual-level ethnic dissimilarity on emotional strain was also included in the model (*c* ’_*w*_-path). Together, these paths represent a 1-1-1 mediation (Preacher et al., 2010).

## Results

### The Relationship Between Team-Level Ethnic Heterogeneity and Individual-Level Ethnic Dissimilarity

As expected, team-level ethnic heterogeneity does not correspond to individual-level ethnic dissimilarity unless the team is completely homogeneous, which was true for eight teams that only consisted of ethnic majority members with a *BI* and *PD* of zero. Moreover, a positive correlation between team-level ethnic heterogeneity and individual-level ethnic dissimilarity is observed only among the ethnic majority, estimate = 0.11, *SE* = 0.03, *p* < .001. For ethnic minority team members, the correlation is negative and non-significant, estimate = −0.08, *SE =* 0.06, *p =* .149. Finally, individual-level ethnic dissimilarity cannot be completely separated from being an ethnic majority or minority or member, *t*(271) = −89.51, *p <* .001, that is, individual-level ethnic dissimilarity is lower for ethnic majority team members (*M* = 0.17, *SD =* 0.13; range = .00 to .58) than for ethnic minority team members (*M* = 0.96, *SD =* 0.06; range = .78 to 1.00) . These are important observations as they show that these variables are not independent of one another.

### Hypothesis Testing

To test our hypotheses, we specified a 2-2-1 mediation and a 1-1-1 mediation in the same multilevel path model (Preacher et al., 2010). Table 2 shows the direct relationships of the multilevel path analyses with random intercepts.

Hypothesis 1 predicted an indirect relationship between high levels of team-level ethnic heterogeneity and high levels of emotional strain via high levels of team-level emotional conflict (2-2-1). Indeed, team-level ethnic heterogeneity predicted high levels of team-level emotional conflict (*a*_*b*_-path). Team-level emotional conflict predicted high levels of emotional strain (*b*_*b*_-path; see Table 2). The mediation explained 54% of the variance in emotional strain, *R*^*2*^ *= .* 54. In line with these relationships, the indirect relationship between team-level ethnic heterogeneity and emotional strain via team-level emotional conflict was positive and significant, estimate = 0.33, *SE = 0.17, p <* .05; 95% MC-CI [0.036, 0.709]. Thus, our results supported Hypothesis 1.

Hypothesis 2 predicted an indirect relationship between high levels of individual-level ethnic dissimilarity and high levels of emotional strain via high levels of individual-level emotional conflict (1-1-1). Contrary to our expectations, individual-level ethnic dissimilarity was associated with *low* levels of individual-level emotional conflict (*a*_*w*_-path). Individual-level emotional conflict was positively related to emotional strain (*b*_*w*_-path; see Table 2). The mediation explained 12% of the variance in emotional strain, *R*^*2*^ = .12. Analyzing the indirect effect showed a significant *negative* relationship between individual-level ethnic dissimilarity and emotional strain via individual-level emotional conflict, estimate = −0.11, *SE* = 0.04, *p* <.01; 95% MC-CI [−0.179, −0.024], Because we found a negative indirect effect, Hypothesis 2 was not supported.

### Robustness Checks

To ensure that our results were not affected by using data from two different measurement points, we tested the hypothesized indirect effects using only variables measured at the first measurement point. These relationships did not differ from the main analysis. Detailed numbers and figures for this robustness check are available on request.

## Discussion

As proposed in our hypotheses, we found that team-level emotional conflict mediated between team-level ethnic heterogeneity and emotional strain, such that high team-level ethnic heterogeneity related to higher levels of emotional strain via high levels of team-level emotional conflict. When team-level ethnic heterogeneity and its impacts on team-level emotional conflict were accounted for, we found that individual-level ethnic dissimilarity was associated with individual-level emotional conflict in a direction opposite of what would be expected: The higher the individual-level ethnic dissimilarity was, the *lower* individual-level emotional conflict (i.e., the individual perception of emotional conflict within the team) was. These findings demonstrate the complexities of ethnic diversity as experienced by individual team members and the importance of considering individual-level ethnic dissimilarity in the context of their team-level ethnic heterogeneity when investigating effects on employee well-being.

### The Effect of Ethnic Dissimilarity on Emotional Conflict in Context

To explore possible explanations for our unexpected finding—high individual-level ethnic dissimilarity accompanied by low individual-level emotional conflict—we consider ethnic majority and minority groups separately in the context of team-level ethnic heterogeneity. Doing so is necessary because group differences are seldom neutral, different reactions to ethnic diversity between ethnic majority and minority group members can be better understood if we recognize social status as an important factor.

### Dynamics in Relatively Homogeneous Teams

Groups with high power and status strive to maintain their status when faced with outgroups, which corresponds with the need to maintain a positively valued distinct social identity through ingroup favoritism and solidarity (Tajfel & Turner, 1986). This need, however, may not be too strong if the group has a clear, dominant majority, as in relatively homogeneous teams dominated by high-status ethnic majority members. The members of the ethnic majority group—in our study, those with German backgrounds for more than two generations—had lower ethnic dissimilarity in general, yet our analysis suggested that they might perceive a *higher* level of emotional conflict within the team. In mostly ethnic majority teams, these ethnic majority members may not need to maintain solidarity among themselves because of their unquestionably dominant presence. As a result, in relatively homogeneous teams, the ethnic majority members may engage in emotional conflict more frequently and perceive them more strongly. Correspondingly, at the individual level, the low ethnic dissimilarity (of the ethnic majority) may be associated with high emotional conflict.

In the same ethnic majority-dominated teams, the few ethnic minority members (i.e., those with very high ethnic dissimilarity) may be more motivated to keep peace with the large ethnic majority group as well as the very small group of people with the same ethnic background (see also Chattopadhyay et al., 2004, 2016). As for perceptions of team-level conflicts, there may be emotional conflict among the ethnic majority members in the team, but the small number of ethnic minority members may not be aware of them. This might be because high-status groups (i.e., the ethnic majority) might perceive disclosing negative personal information to be threatening their status distance (Phillips et al., 2009) and therefore do not communicate about emotional conflict. Also, ethnic minorities are possibly isolated from the rest of the team if they comprise a small share of the overall team (Kanter, 1977). Thus, in teams with low ethnic heterogeneity (i.e., ethnic majority-dominated teams), very high ethnic dissimilarity of ethnic minority members may be associated with low levels of emotional conflict both at the individual and team levels.

### Dynamics in Relatively Heterogeneous Teams

In teams with more heterogeneous ethnic compositions, the dynamics likely differ. Because Germans without migration backgrounds are the numerical majority in general, they have relatively low ethnic dissimilarity even in these teams. They may, however, feel a stronger need to distinguish themselves from the low- status members than their counterparts in more homogeneous, ethnic majority-dominated teams do. They may engage in more emotional conflict with members of lower-status groups. Thus, in more heterogeneous teams, the relatively low ethnic dissimilarity of the ethnic majority may have been associated with more emotional conflict.

As for the ethnic minority members in heterogeneous teams, who had lower ethnic dissimilarity than their colleagues in more homogenous teams but still had high ethnic dissimilarity, their experiences of social processes can be explored from the social mobility perspective. Chattopadhyay et al. (2004) propose that minority group members’ interaction with majority members depends on the perceived permeability of the boundary that separates the groups. If ethnic minority members believe that they can be accepted by the ethnic majority group and therefore enjoy some of the benefits of majority status, they will maintain positive interactions with ethnic majority members and keep their distance from their own or other ethnic minority groups. If they do not believe that the boundary is permeable, they identify more strongly with their own groups and may engage in emotional conflict with ethnic majority members. In our study, these additional dimensions were not measured and thus could not be investigated. However, the explicit focus on ethnic minority members’ perspectives about social boundaries is a promising direction for future research.

In summary, experience with individual-level ethnic dissimilarity needs to be examined within the context of team-level ethnic heterogeneity, the social standing of the group to which each person belongs, and the beliefs and desires team members have about changing their status. This expands the traditional understanding of ethnic diversity from the similarity/attraction paradigm. Investigating ethnic dissimilarity as embedded in a team’s ethnic compositions may also shed light on the asymmetry hypothesis—ethnic minority members may not experience the effects of ethnic diversity the same way that ethnic majority members do (Chattopadhyay, 1999; Hoppe et al., 2014; Riordan, 2000; Tsui et al., 1992)—which has been acknowledged in the relational demography literature. The relationship between the ethnic majority and minority, not only within the workplace but also in society at large, is likely to be reflected in team processes and, ultimately, to influence emotional strain. Future studies with larger samples should also test the asymmetry hypothesis along with both levels of ethnic diversity.

### Practical Implications

As teams and workplaces are becoming more diverse, management and supervisors will need to react to challenges that may arise in ethnically diverse teams such as social conflicts within and across ethnic groups with different social standing. Guillaume et al. (2012) suggest that specifically in ethnically diverse teams, establishing team interdependence is crucial to improve social relationships, for example, by implementing common group tasks or rewards for the team. Furthermore, Liebermann et al. (2013) suggest that ethnic stereotyping within work teams can be reduced by emphasizing similarities between different ethnic groups and creating an atmosphere that enables team members to get to know each other. Diversity and cultural awareness training may help in this regard (see also Brodbeck et al., 2011). Organizations should strive for effective diversity policies that involve an inclusive and diversity-friendly climate (Drach-Zahavy & Trogan, 2013; van Dick et al., 2008).

Our findings showed that among all team members—irrespective of their ethnic background—emotional conflict was related to emotional strain. Interventions at the team and individual level on conflict resolution are likely to be beneficial for health and well-being in all teams (Hyde et al., 2006) but may be even more important in ethnically diverse teams. Finally, enhancing existing social ties or developing new ones in the workplace facilitates social support among colleagues and, in turn, their health and well-being (Heaney, 2017).

### Strengths and Limitations

We studied ethnic diversity effects in real work teams, which provided us with high external validity. These teams were part of a single organization and all members had the same job (i.e., retail store clerks); therefore, the effects of ethnic diversity we found were not blurred by occupational or organizational differences. While this is a strength, a specific sample always limits the generalizability of our findings. As task complexity and team interdependence influence whether ethnic diversity becomes an asset or a liability (e.g., van Knippenberg et al., 2004), our results may be generalizable to teams working with similar levels of interdependence in other pink- or blue-collar jobs (i.e., simple service and manual labor). Findings from studies with nurses and warehouse workers (e.g., Drach-Zahavy & Trogan, 2013; Hoppe et al., 2014) point to similar directions as our findings, but more research across occupations and organizations is needed to generalize these results.

As with most studies on ethnic diversity, the share of ethnic minority members was low in this sample. While the ethnic composition of our sample was roughly proportional to the German workforce, we were able to examine only a limited range of team-level ethnic heterogeneity and unequal ranges of individual-level ethnic dissimilarity between ethnic majority and minority members. Consequently, we could not test the differential effects within the ethnic minority groups. Nonetheless, studying ethnic diversity in less diverse teams is important because this is the reality in many workplaces.

A strength of the paper is the high response rate of 86%. In addition, we had the unique opportunity to use administrative data on the workers’ birthplace and their nationality for missing self-report information on ethnic background (14%). This administrative data enabled us to compute more accurate scores for team-level ethnic heterogeneity and individual-level ethnic dissimilarity based on the ethnic background of all members, regardless of their participation in the study. This is a major improvement from previous studies that used only the data available from self-reports (e.g., Tsui et al., 1992). The administrative data we used, however, identified only the first-generation ethnic minorities but not the second generation. Potentially, we may have underestimated the percentage of ethnic minority workers. However, when comparing the number of ethnic minority workers when using administrative data for missing information versus self-report information only, we do not see differences in the percentage of ethnic minority workers. Finally, our cross-sectional design does not allow causality claims. However, using self-reports from two measurement points enabled us to reduce common method variance (Podsakoff et al., 2003).

## Conclusion

Investigating effects of ethnic diversity on well-being at both the team and individual levels provided us with the insight that ethnic diversity effects may not be the same between the two levels. Our findings suggested intricate dynamics within teams, which may be different for the ethnic majority and minority members of society. In most studies in the diversity literature, ethnic majority members account for the greatest proportion in study samples; therefore, the current findings are, unwittingly, about the ethnic majority’s reactions to the presence of ethnic minorities and overlook ethnic minority members’ reactions to ethnic diversity. Team members’ reactions to others who are in some way different from themselves are complex and need to be explored more carefully in contexts, both in the workplace and society at large. A first step to creating a more complete picture is to study individual-level ethnic dissimilarity in the context of team-level ethnic heterogeneity with an explicit focus on power and status dynamics among groups.

## Figures and Tables

**Table 1:** Means (*M*), Standard Deviations (*SD*), Cronbach’s Alphas (α), Test-Retest Reliabilities (*r*_*tt*_), Intraclass Correlations 1 (ICC), and Intercorrelations for Study Variables

	*M*	*SD*	*r*_*tt*_	α	ICC	1	2	3	4	5
1. Team-level ethnic heterogeneity	0.29	0.20								
2. Team-level emotional conflict	2.09	0.46	.77			.30*				
3. Emotional strain ^a^	2.06	0.38	.35			−.16	.26			
4. Individual-level ethnic dissimilarity	0.29	0.32								
5. Individual-level emotional conflict	2.13	0.92	.74	.86	.20				.03	
6. Emotional strain ^a^	2.13	0.90	.71	.88	.04				−.08	.35***

*Note.* Team-level intercorrelations are in the upper half of the Table *(N=*50). Individual-level inter-correlations are in the lower half of the table (*n*= 602). The information for both team- and individual-level emotional conflict stems from measurement point 1; the information for emotional strain stems from measurement point 2

a Because the main analysis decomposes the variance and uses team variance in relationships with team-level variables, we show emotional strain in line 3 as team-level aggregate and line 6 as individual-level variable without aggregation

* *p* <.05.

*** *p* <.001

**Table 2:** Unstandardized Estimates (*B*), Including Standard Errors (*SE E*), and Standardized Estimates (γ) of the Multilevel Path Analysis

	*B*	*SE B*	γ
*Team level*			
*a*_*b*_-path: ethnic heterogeneity → emotional conflict	1.01**	0.35	.48
*b*_*b*_-path: emotional conflict → emotional strain	0.33*	0.14	.83
*c* ‘_*b*_-path: ethnic heterogeneity → emotional strain	−0.40	0.33	−.48
Intercept emotional conflict	1 89***	0.12	
Intercept emotional strain	1.56***	0.27	
Residual variance emotional conflict	0.13***	0.03	
Residual variance emotional strain	0.01	0.01	
*Individual level*			
*a*_*w*_-path: ethnic dissimilarity → emotional conflict	−0.29*	0.12	−.11
*b*_*w*_-path: emotional conflict → emotional strain	0.36***	0.04	.34
*c* ‘_*w*_-path: ethnic dissimilarity → emotional strain	−0.08	0.21	−.03
Residual variance emotional conflict	0.70***	0.06	
Residual variance emotional strain	0.69***	0.05	

*Note.* These models are a 2-2-1 and a 1-1-1 mediation model with random intercepts. All paths are fixed The subscript_*b*_ indicates a path at the between-team level; the subscript_*w*_ indicates a path at the within-team level

* *p* <.05.

** *p* <.01.

*** *p* <.001

## Data Availability

The works council of the participating company voted against data sharing to protect their workers’ privacy.

## Footnotes

1 By ethnic minority members, we refer to people with an ethnic background that is not German, and by ethnic majority members, we refer to people with a German ethnic background.

2 We excluded teams that were involved in this pre-exploration from the final sample.

3 As 4% of the team members did not provide complete information on parents’ ethnic background, we had to derive this information from administrative data as described on p.16.

4 We deviated slightly from the original scale by using statements instead of questions in order to offer the same response options as for emotional strain.

5 Both predictors were assumed to be stable over the one-month assessment time. We used the measure of emotional conflict from the first measurement point and emotional strain from the second.

## References

[R1] Agresti A, & Agresti BF (1978). Statistical analysis of qualitative variation. Sociological Methodology, 9, 204–237. 10.2307/270810

[R2] Allen NJ, Stanley DJ, Williams HM, & Ross SJ (2007). Assessing the impact of nonresponse on work group diversity effects. Organizational Research Methods, 10(2), 262–286. 10.1177/1094428106/294731

[R3] Berg M, Schor P, & Soto I (2014). The weight of words: Writing about race in the United States and Europe. American Historical Review, 119(3), 800–808. 10.1093/ahr/119.3.800

[R4] Blau PM (1977). Inequality and heterogeneity: A primitive theory of social structure. Free Press.

[R5] Bliese PD (2000). Within-group agreement, non-independence, and reliability. Implications for data aggregation and analysis. In Klein KJ & Kozlowski SWJ (Eds.), Multilevel theory, research, and methods in organizations (pp. 349–381). Jossey-Bass.

[R6] Brewer MB (1979). In-group bias in the minimal intergroup situation: A cognitive-motivational analysis. Psychological Bulletin, 86(2), 307–324. 10.1037/0033-2909.86.2307

[R7] Brodbeck FC, Guillaume YRF, & Lee NJ (2011). Ethnic diversity as a multilevel construct: The combined effects of dissimilarity, group diversity, and societal status on learning performance in work groups. Journal of Cross-Cultural Psychology, 42(1), 1198–1218. 10.1177/0022022110383314

[R8] Byrne D (1971). The attraction paradigm. Academic Press.

[R9] Chan D (1998). Functional relations among constructs in the same content domain at different levels of analysis: A typology of composition models. Journal of Applied Psychology, 83(2), 234–246. 10.1037/0021-9010.83.2.234

[R10] Chatman JA, & Flynn FJ (2001). The influence of demographic heterogeneity on the emergence and consequences of cooperative norms in work teams. Academy of Management Journal, 44(5), 956–974. 10.5465/3069440

[R11] Chattopadhyay P (1999). Beyond direct and symmetrical effects: The influence of demographic dissimilarity on organizational citizenship behavior. Academy of Management Journal, 42(3), 273–287. 10.5465/256919

[R12] Chattopadhyay P, Tluchowska M, & George E (2004). Identifying the ingroup: A closer look at the influence of demographic dissimilarity on employee social identity. The Academy of Management Review, 29(2), 180–202. 10.2307/20159028

[R13] Chattopadhyay P, George E, & Ng CK (2016). Hearts and minds. Organizational Psychology Review, 6(2), 119–144. 10.1177/2041386615574540

[R14] Constant AF, Nottmeyer O, & Zimmermann KF (2012). Cultural integration in Germany. In Algan Y, Bisin A, Manning A, & Verdier T (Eds.), Cultural integration of immigrants in Europe (pp. 69–124). Oxford University Press. 10.1093/acprof:oso/9780199660094.003.0003

[R15] De Dreu CKW, & Beersma B (2005). Conflict in organizations: Beyond effectiveness and performance. European Journal of Work and Organizational Psychology, 14(2), 105–117. 10.1080/13594320444000227

[R16] De Dreu CKW, & Weingart LR (2003). Task versus relationship conflict, team performance, and team member satisfaction: A meta-analysis. Journal of Applied Psychology, 88(4), 741–749. 10.1037/0021-9010.88.4.741 12940412

[R17] de Wit FRC, Greer LL, & Jehn KA (2012). The paradox of intragroup conflict: A meta-analysis. Journal of Applied Psychology, 97(2), 360–390. 10.1037/a0024844 21842974

[R18] Dormann C, & Zapf D (2002). Social stressors at work, irritation, and depressive symptoms: Accounting for unmeasured third variables in a multi-wave study. Journal of Occupational and Organizational Psychology, 75(1), 33–58. 10.1348/096317902167630

[R19] Drach-Zahavy A, & Trogan R (2013). Opposites attract or attack? The moderating role of diversity climate in the team diversity-interpersonal aggression relationship. Journal of Occupational Health Psychology, 18(A), 449–457. 10.1037/a0033989 24099164

[R20] Guillaume YRF, Brodbeck FC, & Riketta M (2012). Surface- and deep-level dissimilarity effects on social integration and individual effectiveness related outcomes in work groups: A meta-analytic integration. Journal of Occupational and Organizational Psychology, 85(1), 80–115. 10.111l/j.2044-8325.2010.02005.x

[R21] Hansen R (2003). Migration to Europe since 1945: Its history and its lessons [Special issue]. The Political Quarterly, 74(s1), 25–38. 10.llll/j.1467-923X.2003.00579.x

[R22] Harrison DA, & Klein KJ (2007). What’s the difference? Diversity constructs as separation, variety, or disparity in organizations. Academy of Management Review, 32(4), 1199–1228. 10.5465/AMR.2007.26586096

[R23] Hartmann J (2016). Do second-generation Turkish migrants in Germany assimilate into the middle class? Ethnicities, 16(3), 368–392. 10.1177/1468796814548234

[R24] Harvey SB, Modini M, Joyce S, Milligan-Saville JS, Tan L, Mykletun A, Bryant RA, Christensen H, & Mitchell PB (2017). Can work make you mentally ill? A systematic meta-review of work-related risk factors for common mental health problems. Occupational and Environmental Medicine, 74(A), 301–310. 10.1136/oemed-2016-104015 28108676

[R25] Hatfield E, Cacioppo JT, & Rapson RL (1994). Emotional contagion. Cambridge University Press. 10.1017/CB09781139174138.

[R26] Heaney CA (2017). Social relationships: Harnessing their potential to promote health. In O’Donnell MP (Ed.), Health promotion in the workplace (5th ed., pp. 615–634). Art & Science of Health Promotion Institute.

[R27] Hoppe A (2011). Psychosocial working conditions and well-being among immigrant and German low- wage workers. Journal of Occupational Health Psychology, 16(2), 187–201. 10.1037/a0021728 21244167

[R28] Hoppe A, Fujishiro K, & Heaney CA (2014). Workplace racial/ethnic similarity, job satisfaction, and lumbar back health among warehouse workers: Asymmetric reactions across racial/ethnic groups. Journal of Organizational Behavior, 35(2), 172–193. 10.1002/job.1860

[R29] Hyde M, Jappinen P, Theorell T, & Oxenstiema G (2006). Workplace conflict resolution and the health of employees in the Swedish and Finnish units of an industrial company. Social Science and Medicine, 63(8), 2218–2227. 10.1016/j.socscimed.2006.05.002 16782255

[R30] International Labour Organization. (2017). ILO global estimates on international migrant workers. Results and methodology. Executive summary. https://bit.ly/2HhOPTa. Accessed 16 July 2020.

[R31] Jehn KA (1994). Enhancing effectiveness: An investigation of advantages and disadvantages of value-based intragroup conflict. International Journal of Conflict Management, 5(3), 223–238. 10.1108/eb022744

[R32] Jehn KA, & Mannix EA (2001). The dynamic nature of conflict: A longitudinal study of intragroup conflict and group performance. Academy of Management Journal, 44(2), 238–251. 10.5465/3069453

[R33] Jehn KA, Chadwick C, & Thatcher SMB (1997). To agree or not to agree: The effects of value congruence, individual demographic dissimilarity, and conflict on workgroup outcomes. International Journal of Conflict Management, 8(4), 287–305. 10.1108/eb022799

[R34] Joshi A, Liao H, & Roh H (2011). Bridging domains in workplace demography research: A review and reconceptualization. Journal of Management, 37(2), 521–552. 10.1177/0149206310372969

[R35] Kanter RM (1977). Some effects of proportions on group life: Skewed sex ratios and responses to token women. American Journal of Sociology, 82(5), 965–990. 10.1086/226425

[R36] Klein K, & Kozlowski S (2000). A multilevel approach to theory and research in organizations: Contextual, temporal, and emergent processes. In Klein K & Kozlowski S (Eds.), Multilevel theory, research, and methods in organizations: Foundations, extensions, and new directions (pp. 3–90). Jossey-Bass. 10.2307/3094811.

[R37] Leonard JS, & Levine DI (2006). The effect of diversity on turnover: A large case study. Industrial and Labour Relations Review, 59(4), 547–572. 10.1177/001979390605900402

[R38] Leon-Perez JM, Antino M, & Leon-Rubio JM (2016). The role of psychological capital and intragroup conflict on employees’ burnout and quality of service: A multilevel approach. Frontiers in Psychology, 7, 1755. 10.3389/fpsyg.2016.01755 27895601 PMC5107570

[R39] Liebermann SC, Wegge J, Jungmann F, & Schmidt K-H (2013). Age diversity and individual team member health: The moderating role of age and age stereotypes. Journal of Occupational and Organizational Psychology, 86(2), 184–202. 10.llll/joop.12016

[R40] Meyer B (2017). Team diversity. In Salas E, Rico R, & Passmore J (Eds.), The Wiley Blackwell handbook of the psychology of team working and collaborative processes (pp. 151–175). Wiley. 10.1002/9781118909997.ch7

[R41] Mohr G, Müller A, Rigotti T, Aycan Z, & Tschan F (2006). The assessment of psychological strain in work contexts: Concerning the structural equivalency of nine language adaptations of the irritation scale. European Journal of Psychological Assessment, 22(3), 198–206. 10.1027/1015-5759.22.3.198

[R42] Ohliger R (2008). Country report on ethnic relations: Germany. EDUMIGROM Background Papers. https://lmy.de/r40Il. Accessed 10 July 2020.

[R43] Pelled LH, Eisenhardt KM, & Xin KR (1999). Exploring the black box: An analysis of work group diversity, conflict, and performance. Administrative Science Quarterly, 44(1), 1–28. 10.2307/2667029

[R44] Phillips KW, Rothbard NP, & Dumas TL (2009). To disclose or not to disclose? Status distance and self-disclosure in diverse environments. Academy of Management Review, 34(4), 710–732. 10.5465/AMR.2009.44886051

[R45] PodsakofF PM, MacKenzie SB, Lee JY, & PodsakofF NP (2003). Common method biases in behavioral research: A critical review of the literature and recommended remedies. Journal of Applied Psychology, 88(5), 879–903. 10.1037/0021-9010.88.5.879 14516251

[R46] Preacher KJ, & Selig JP (2012). Advantages of Monte Carlo confidence intervals for indirect effects. Communication Methods and Measures, 6(2), 77–98. 10.1080/19312458.2012.679848

[R47] Preacher KJ, Zyphur MJ, & Zhang Z (2010). A general multilevel SEM framework for assessing multilevel mediation. Psychological Methods, 15(3), 209–233. 10.1037/a0020141 20822249

[R48] Prüfer P, & Rexroth M (2005). Cognitive interviews. In GESIS - How to (Vol. 15). DEU. https://www.ssoar.info/ssoar/handle/document/20147. Accessed 27 November 2018.

[R49] Riordan CM (2000). Relational demography within groups: Past developments, contradictions, and new directions. In Ferris GR (Ed.), Research in personnel and human resources management (Vol. 19, pp. 131–173). JAI Press. 10.1016/S0742-7301(00)19005-X.

[R50] Riordan CM, & Shore LM (1997). Demographic diversity and employee attitudes: An empirical examination of relational demography within work units. Journal of Applied Psychology, 82(3), 342–358. 10.1037/0021-9010.82.3.342

[R51] Riordan CM, & Wayne JH (2008). A review and examination of demographic similarity measures used to assess relational demography within groups. Organizational Research Methods, 77(3), 562–592. 10.1177/1094428106295503

[R52] Schäfer H, & Schmidt J (2016). Beschäftigung im Einzelhandel [Employement in retail trade]. Institut der deutschen Wirtschaft Köln. https://bit.ly/3nck7vD. Accessed 29 June 2019.

[R53] Selig JP, & Preacher KJ (2008). Monte Carlo method for assessing mediation: An interactive tool for creating confidence intervals for indirect effects [Computer software]. http://www.quantpsy.org/medmc/medmc.htm. Accessed 30 July 2018.

[R54] Spector PE, & Bruk-Lee V (2008). Conflict, health, and well-being. In De Dreu CKW & Gelfand MJ (Eds.), The psychology of conflict and conflict management in organizations (pp. 267–288). Lawrence Erlbaum Associates.

[R55] Spector PE, & Jex SM (1998). Development of four self-report measures of job stressors and strain: Interpersonal conflict at work scale, organizational constraints scale, quantitative workload inventory, and physical symptoms inventory. Journal of Occupational Health Psychology, 5(4), 356–367. 10.1037/1076-8998.3.4.356 9805281

[R56] Statistisches Bundesamt. (2018). Bevölkerung und Erwerbstätigkeit: Bevölkerung mit Migrationshintergrund, Ergebnisse des Mikrozensus 2017 [Population and employment: Population with migration background, results of the microcensus 2017] (Vol. 1, Issue 2.2). https://bit.ly/3plWlB5. Accessed 15 September 2019.

[R57] Tajfel H, & Turner JC (1986). The social identity theory of intergroup behavior. In Worchel S & Austin WG (Eds.), Psychology of intergroup relations (2nd ed., pp. 7–24). Nelson-Hall.

[R58] Tjosvold D (2008). The conflict-positive organization: It depends upon us. Journal of Organizational Behavior, 29(1), 19–28. 10.1002/job.473

[R59] Tsui AS, Egan TD, & O’Reilly CA (1992). Being different: Relational demography and organizational attachment. Administrative Science Quarterly, 57(4), 549–579. 10.2307/2393472

[R60] Turner JC, Hogg MA, Oakes PJ, Reicher SD, & Wetherell MS (1987). Rediscovering the social group: A self-categorization theory. Basil Blackwell.

[R61] United Nations, Department of Economic & Social Affairs, Population Division (2018). International Migration Report 2017 (ST/ESA/SER.A/404). https://bit.ly/3posjeN. Accessed 2 June 2020.

[R62] van Dick R, van Knippenberg D, Hägele S, Guillaume YRF, & Brodbeck FC (2008). Group diversity and group identification: The moderating role of diversity beliefs. Human Relations, 67(10), 1463–1492. 10.1177/0018726708095711

[R63] van Knippenberg D, De Dreu CKW, & Homan AC (2004). Work group diversity and group performance: An integrative model and research agenda. Journal of Applied Psychology, 89(6), 1008–1022. 10.1037/0021-9010.89.6.1008 15584838

[R64] Williams HM, & Meân LJ (2004). Measuring gender composition in work groups: A comparison of existing methods. Organizational Research Methods, 7(4), 456–474. 10.1177/1094428104269175

[R65] Zhang Z, Zyphur MJ, & Preacher KJ (2009). Testing multilevel mediation using hierarchical linear models. Organizational Research Methods, 12(4), 695–719. 10.1177/1094428108327450

